# Exploring the interconnectedness of multiple sclerosis and erectile dysfunction: mechanisms and management strategies

**DOI:** 10.3389/fcell.2025.1728027

**Published:** 2025-12-10

**Authors:** Yongrui Zhang, Hongliang Cao, Lei Wang, Tao Xu, Bo Yuan

**Affiliations:** Department of Urology II, The First Hospital of Jilin University, Changchun, China

**Keywords:** multiple sclerosis, erectile dysfunction, neurogenic bladder, urodynamics, neuromodulation

## Abstract

Erectile dysfunction (ED) is common in men with multiple sclerosis (MS) and arises from convergent neurogenic, autonomic, vascular, inflammatory, and psychosocial pathways. We synthesize epidemiologic prevalence and risk data on ED in MS, map mechanistic pathways linking central and spinal lesions, autonomic–endothelial imbalance, and psychosocial modulators to ED phenotypes, and provide a stage-based care algorithm integrating neuro-urological testing and psycho-sexological support. Across more than 30 studies, pooled ED prevalence in men with MS is approximately 50%, with higher odds at greater disability and with co-existing lower urinary tract symptom (LUTS) and depression. Phosphodiesterase-5 inhibitors (PDE5i) are commonly used as first-line pharmacologic therapy, although data specific to MS remain limited and individual response can vary. Device-, injection-, neuromodulation-, and prosthesis-based options offer escalation pathways. Evidence quality varies and residual confounding and detection bias remain possible.

## 1 Introduction

Erectile dysfunction (ED) is defined as the persistent inability to attain or maintain a penile erection sufficient for satisfactory sexual performance (Shamloul and Ghanem, 2013). Clinically, ED is commonly categorized as psychogenic, organic (neurogenic, vasculogenic, endocrinologic, or medication-induced), or mixed in origin, with substantial overlap in real-world settings (Glina et al., 2014; Thomas et al., 2021). Over recent decades, the global burden of ED has risen in parallel with population aging, cardiometabolic comorbidities, and survivorship after chronic diseases, reflecting both improved recognition and genuine epidemiologic growth (Randrup et al., 2015; Rew and Heidelbaugh, 2016). Beyond impairing sexual health and quality of life for patients and partners, ED imposes significant health-economic pressures through increased healthcare utilization, productivity loss, and downstream cardiovascular and mental health sequelae (Liu et al., 2018; Rezaee et al., 2020). Current diagnostic practice relies on focused sexual history, validated questionnaires, and selective laboratory or physiological testing to phenotype causes and guide therapy (Glina et al., 2014; Rew and Heidelbaugh, 2016). First-line management centers on lifestyle optimization and phosphodiesterase-5 inhibitors (PDE5i), with adjunctive psychosocial interventions, pelvic floor rehabilitation, devices, or surgical options for nonresponders (Mitidieri et al., 2020; Köhler et al., 2024). However, gaps persist: heterogeneous assessment standards, under-screening in high-risk groups, limited integration of neuro-urological evaluation, inconsistent attention to comorbidities and medications, and scarce mechanism-informed, personalized strategies. Preventive frameworks remain underdeveloped, and longitudinal data linking targeted interventions to durable sexual and overall health outcomes are limited, underscoring the need for more rigorous, multidisciplinary research (Wang et al., 2023; Hentzen et al., 2022).

Multiple sclerosis (MS) is a chronic, immune-mediated demyelinating disease of the central nervous system characterized by inflammatory lesions, axonal loss, and progressive neurodegeneration (Multiple sclerosis, 2018; Oh et al., 2018). Current MS phenotypes include: relapsing-remitting MS (RRMS), clinically isolated syndrome (CIS), radiologically isolated syndrome (RIS), primary-progressive MS (PPMS), and secondary-progressive MS (SPMS) (Kat and z Sand, 2015). MS is tightly interconnected with disorders across multiple organ systems, including mood and anxiety disorders, sleep disturbances, chronic pain syndromes, autonomic dysfunction with lower urinary tract symptoms, bowel dysfunction, osteoporosis, metabolic syndrome, cardiovascular disease, sexual dysfunctions, and treatment-related endocrine and hematologic effects (Kat and z Sand, 2015; The Lancet, 2021; Marcus, 2022; Nesbitt et al., 2024; Doshi and Chataway, 2016; Correale et al., 2017). Emerging studies increasingly indicate a close relationship between MS and the onset, persistence, and progression of ED, implicating convergent neurogenic, vascular, autonomic, inflammatory, and psychosocial pathways (Shaygannejad et al., 2025; Yazdan Panah et al., 2025; Giannopapas et al., 2023; Çıracıoğlu et al., 2025; Redelman, 2009). Deepening our understanding of these links offers a fresh vantage point for prevention, early detection, and tailored management. This review synthesizes epidemiology and risk correlates of erectile dysfunction in men with multiple sclerosis, explains mechanistic pathways linking MS pathology to ED, proposes a stage-based clinical algorithm, evaluates current therapies and escalation strategies, and highlights measurement considerations, research gaps, and implementation priorities.

## 2 Observational evidences for a robust MS–ED association

A growing body of observational research—spanning population-based datasets, case–control comparisons, cross-sectional cohorts, and systematic reviews/meta-analyses—demonstrates a consistent, clinically meaningful association between MS and ED (Table 1). Early clinic-based series already reported high rates of sexual dysfunction in men with MS, with erectile failure predominating and frequently coexisting with neuro-urological symptoms and pyramidal signs, suggesting neurogenic underpinnings proximal to the sacral cord (Betts et al., 1994; Valleroy and Kraft, 1984; Kessler et al., 2009). Early case–control work also suggested a higher risk of sexual dysfunction in MS compared with controls, providing historical context for later observational reports (Zorzon et al., 1999). Subsequent physiologic studies corroborated frequent abnormalities in genital somatosensory pathways despite preserved nocturnal erections in a subset, supporting central afferent pathway disruption as a contributor to MS-related ED (Yang et al., 2001; Kirkeby et al., 1988a; Kirkeby et al., 1988b). Voxel-wise lesion mapping further linked ED deterioration to juxtacortical insular lesions—a hub activated during sexual arousal—independent of age, disease duration, depression, or total lesion volume (Winder et al., 2018).

**TABLE 1:** Summary of representative observational evidence linking MS to ED.

Study	Design/Population	Key Measures	Main Findings
Zorzon et al. (1999)	Case–control	ED	Higher prevalence of sexual dysfunction in MS versus controls, supporting increased population-level risk.
Wu et al. (2022)	Systematic review/meta-analysis; 16 studies; 2,760 men with MS	Pooled prevalence; RR vs. controls	Pooled ED prevalence ≈49%; MS associated with higher ED risk (RR 3.17; 95% CI 2.31–4.36); subgroup trends with age, IIEF use, longer disease duration
Shaygannejad et al. (2025)	Systematic review/meta-analysis; 29 studies; 3,349 men with MS	Pooled prevalence	ED prevalence ≈49% (95% CI 47%–50%); SD overall ≈66%
Keller et al. (2012)	Nationwide case–control (Taiwan NHIRD); 38,139 ED cases, 262,848 controls	Adjusted OR for prior MS	Higher odds of prior MS among ED cases (aOR 2.23; 95% CI 1.15–4.32)
Balsamo et al. (2017)	Cross-sectional; 101 men with MS	IIEF-15, SQoL-M, IPSS, BDI-II, EDSS, urodynamics	ED in 74%; depression and LUTS independently predicted ED; associations with EDSS and detrusor underactivity on univariate analysis
Fragalà et al. (2015)	Cross-sectional; 135 MS with LUTD	Urodynamics, IIEF-15/FSFI, EDSS	PdetmaxIDC ≥20 cmH2O, MCC <135 mL, and compliance ≤3 mL/cmH2O predicted moderate–severe ED; links ED with neurogenic bladder physiology
Winder et al. (2018)	MRI lesion-symptom mapping; 31 men with MS	ΔIIEF-5, voxel-wise lesion analysis	ED deterioration associated with bilateral (predominantly left) insular juxtacortical lesions; not explained by age, duration, depression, or total lesion volume
Nabavi et al. (2021)	Multicenter cross-sectional; 320 men with MS (Iran)	IIEF, MSISQ-19, SQOL-M, GHQ, EDSS	SD prevalence 35.6% (IIEF ≤45); independent predictors: age, MSISQ-19, SQOL-M; univariate: EDSS, duration, mood, smoking
Lew-Starowicz and Rola (2014a)	Cross-sectional; 204 MS patients	IIEF/FSFQ, SQoL, BDI, EDSS	Depression common (≈52%); sexual domains correlated with depressive symptoms and brainstem signs; SQoL negatively impacted
Lew-Starowicz and Rola (2014b)	Cross-sectional; 67 men with MS	IIEF, SQoL, EDSS	ED 53%; strong impact on SQoL; poor disclosure and low care engagement
Bientinesi et al. (2022)	Prospective single cohort; 57 men with MS	IIEF-5, ICIQ-MLUTS, EDSS, Dyadic Adjustment Scale	Worse marital adjustment associated with lower IIEF-5, higher LUTS, higher EDSS; underscores psychosocial burden
Adamec et al. (2024)	Multicenter observational cohort	IIEF-5, PEDT	Disability (EDSS) independently associated with lower IIEF domains after multivariable adjustment

(1) ED, definitions and thresholds vary across studies (e.g., IIEF-5, vs. IIEF-15, domains); prevalences are not strictly comparable across instruments. (2) Meta-analytic risk estimates (Wu et al., 2022) adjust for study-level differences; heterogeneity for risk estimate was minimal, but prevalence heterogeneity was high. (3) Case–control OR (Keller et al., 2012) reflects the odds of prior MS, among men with ED; temporality cannot be inferred. (4) Urodynamic predictors (Fragalà et al., 2015) indicate pathophysiological links to neurogenic bladder rather than proof of causality. (5) Psychological comorbidity (e.g., depression) commonly co-segregates with ED, in MS, potentially mediating part of the association.

Abbreviation: MS, multiple sclerosis; ED, erectile dysfunction; RR, relative risk; CI, confidence interval; aOR, adjusted odds ratio; IIEF, international index of erectile function; IIEF-5, international index of erectile function-5; IIEF-15, international index of erectile function-15; SQoL-M, Sexual quality of life–male; IPSS, international prostate symptom score; BDI-II, beck depression inventory-II; EDSS, expanded disability status scale; LUTS, lower urinary tract symptoms; LUTD, lower urinary tract dysfunction; FSFI, female sexual function index; PdetmaxIDC, maximum detrusor pressure at involuntary detrusor contraction; MCC, maximum cystometric capacity; MRI, magnetic resonance imaging; NPT, nocturnal penile tumescence; SEP, somatosensory evoked potential; BCR, bulbocavernosus reflex; GHQ, general health questionnaire; MSISQ-19, Multiple sclerosis intimacy and sexuality questionnaire-19.

At the epidemiologic level, two complementary meta-analyses converge on a high prevalence of ED among men with MS and a significantly elevated relative risk versus non-MS populations. In pooled analyses, the prevalence of ED among men with MS is approximately one in two, with subgroup trends suggesting higher burden with older age, longer disease duration, and when diagnosed using IIEF-based instruments (Shaygannejad et al., 2025; Wu et al., 2022). Importantly, Wu et al. estimated at more than threefold increased risk of ED in MS relative to controls with negligible between-study heterogeneity in risk estimates, reinforcing the robustness of the association (Wu et al., 2022). Population-scale case–control data from Taiwan also showed higher odds of prior MS among men diagnosed with ED after adjustment for key cardiometabolic and sociodemographic confounders (Keller et al., 2012), extending generalizability beyond single-center cohorts.

Clinic-based cross-sectional studies increasingly suggest both high ED frequency and its correlation with MS-related disability and co-symptomatology. In an Italian multi-institutional cohort, ED (IIEF-EF ≤25) affected roughly three-quarters of male patients and was associated in multivariable analyses with depressive burden and lower urinary tract symptom (LUTS) severity, underscoring the interplay between neuro-urological and psychosocial domains (Balsamo et al., 2017). In a hospital MS cohort, ∼70% reported at least one sexual dysfunction, with common male issues including ED and altered ejaculation; about 22% experienced frequent problems. Longer disease duration correlated with lower sexual satisfaction, while lack of sexual interest was most prevalent and negatively correlated with disability (EDSS), underscoring the need for routine SD/ED screening and management in MS (Calabrò et al., 2018). This gradient has been replicated in a recent multicenter cohort, where disability severity (EDSS) showed consistent, domain-specific declines in erectile function and remained independently associated after adjustment for age and disease duration (Adamec et al., 2024). Studies focused on MS cohorts with lower urinary tract dysfunction (LUTD) found that urodynamic markers of neurogenic bladder—higher involuntary detrusor pressure, low compliance, and reduced cystometric capacity—were independent predictors of moderate–severe ED, linking ED severity to objective LUTD (Fragalà et al., 2014; Fragalà et al., 2015). Other multicenter and single-center analyses similarly associate worse ED/sexual function with higher EDSS, longer disease duration, and coexisting depression and fatigue, while also documenting substantial underreporting and care gaps (Nabavi et al., 2021; Odabaş et al., 2018; Lew-Starowicz and Rola, 2014a; Lew-Starowicz and Rola, 2014b). Notably, marital relationship and quality-of-life measures correlate inversely with ED and LUTS severity, highlighting broader psychosocial impacts within affected couples (Bientinesi et al., 2022). In a cohort of 221 MS patients (124 men, 97 women), male ED was common (≈45% with mild–severe IIEF impairment), often accompanied by decreased libido; among those opting treatment, sildenafil 50–100 mg improved IIEF Q3–Q4 (erection achievement/maintenance) and patient-reported sexual quality of life, with good safety (Dachille et al., 2008).

Synthesizing the available observational literature, a coherent picture emerges that men with MS experience a substantially greater burden of ED than their counterparts without MS, a pattern that recurs across clinic-based cohorts, population datasets, and meta-analytic summaries (Shaygannejad et al., 2025; Wu et al., 2022; Keller et al., 2012). This epidemiologic signal aligns with biologically plausible pathways: lesion-symptom mapping implicates juxtacortical insular and suprasacral regions integral to sexual arousal and autonomic control, while neurophysiological assessments frequently reveal disturbances in genital somatosensory conduction; complementary urodynamic findings further anchor erectile impairment to objective markers of neurogenic lower urinary tract dysfunction (Betts et al., 1994; Yang et al., 2001; Winder et al., 2018; Fragalà et al., 2015). Clinically, ED severity tends to track with greater neurological disability and longer disease duration and is compounded by mood disorders and lower urinary tract symptoms, with downstream repercussions for relationship quality and overall wellbeing (Balsamo et al., 2017; Nabavi et al., 2021; Lew-Starowicz and Rola, 2014a; Lew-Starowicz and Rola, 2014b; Bientinesi et al., 2022). Collectively, these strands converge on a robust, clinically meaningful MS–ED association that warrants systematic recognition and targeted management.

While most available studies are observational and cross-sectional—limiting causal inference and temporal sequencing—the convergence of epidemiologic signals, imaging/physiologic correlates, and dose–response relationships with disability and neuro-urological dysfunction collectively argues for a genuine, clinically relevant linkage. Taken together, these data support systematic screening in MS clinics and provide a rationale for mechanism-informed management, while causality remains to be established in prospective designs.

## 3 Potential mechanistic pathways linking MS to ED

### 3.1 Central arousal networks and limbic–autonomic integration

**FIGURE 1:**
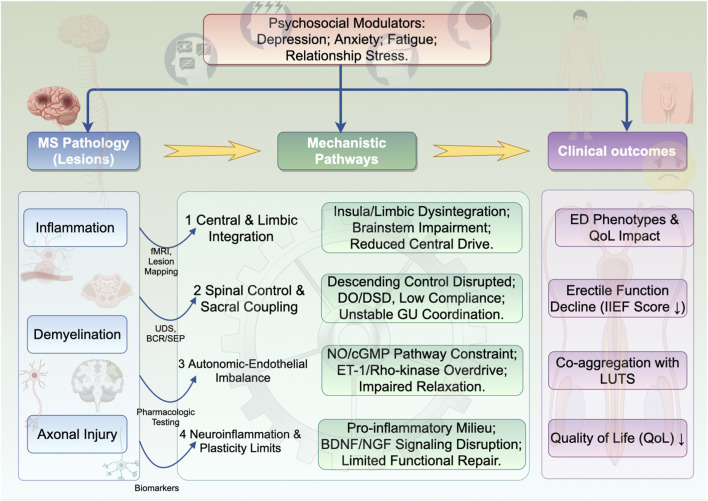
Mechanistic pathways linking MS to ED: an integrative framework. MS pathology (inflammation, demyelination, and axonal injury) converges on four primary mechanistic pathways: (1) impaired central arousal and limbic-autonomic integration, (2) disrupted supraspinal-spinal control and sacral genitourinary coupling, (3) autonomic-endothelial imbalance within the cavernosal tissue, and (4) neuroinflammation-limited trophic plasticity. Psychological and relational factors (e.g., depression, anxiety, fatigue) act as critical modulators, operating under a neurogenic ceiling to shape the final clinical expression. These pathways culminate in heterogeneous ED phenotypes, which often co-aggregate with lower urinary tract symptoms (LUTS) and significantly impact quality of life. Key evidence supporting each pathway (e.g., lesion-symptom mapping, urodynamics, pharmacologic responses) is noted. Abbreviations: ACC, anterior cingulate cortex; BCR, bulbocavernosus reflex; BDNF, brain-derived neurotrophic factor; cGMP, cyclic guanosine monophosphate; DO, detrusor overactivity; DSD, detrusor-sphincter dyssynergia; ED, erectile dysfunction; eNOS, endothelial nitric oxide synthase; ET-1, endothelin-1; fMRI, functional magnetic resonance imaging; IIEF, international index of erectile function; LUTS, lower urinary tract symptoms; MS, multiple sclerosis; NGF, nerve growth factor; NO, nitric oxide; QoL, quality of life; SEP, somatosensory evoked potentials; Trk, tropomyosin receptor kinase; UDS, urodynamic studies.

Data from lesion–symptom mapping, neurophysiology, and clinical cohorts support a central mechanism in which MS lesions disrupt networks that integrate interoception, salience, and autonomic output relevant to sexual arousal. Voxel-wise analyses link declines in erectile function to juxtacortical insular lesions—predominantly left-sided—with effects persisting after adjustment for age, disease duration, depression scores, and total lesion load, indicating a region-specific contribution rather than a mere proxy for global disease severity (Winder et al., 2018). Early and later neurophysiological series demonstrate abnormalities of genital somatosensory evoked potentials and bulbocavernosus reflexes in men with MS and ED, often with partial preservation of nocturnal penile tumescence, suggesting impaired afferent conduction and cortical integration alongside residual efferent capacity (Valleroy and Kraft, 1984; Yang et al., 2001; Kirkeby et al., 1988a; Kirkeby et al., 1988b). Clinically, sexual dysfunction clusters with brainstem and pyramidal signs, and higher disability aligns with worse erectile indices across cohorts, consistent with involvement of limbic–prefrontal–brainstem circuits that modulate both psychogenic and reflexogenic arousal (Betts et al., 1994; Adamec et al., 2024; Lew-Starowicz and Rola, 2014a; Lew-Starowicz and Rola, 2014b; Hennessey et al., 1999). These observations together delineate a central mechanism whereby demyelinating injury within insular–limbic hubs lower autonomic drive to erection and blunts the integration of genital afferent input, constraining arousal despite variably preserved downstream generators (Figure 1).

### 3.2 Descending spinal control and sacral neuro-urological coupling

MS frequently affects suprasacral tracts that coordinate sacral parasympathetic and somatic nuclei, yielding a coupled neurogenic bladder–erectile phenotype. Classic and contemporary neuro-urology work localizes the dominant dysfunction proximal to the sacral cord based on the frequent co-occurrence of detrusor overactivity, detrusor–sphincter dyssynergia, and ED in men with pyramidal signs (Betts et al., 1994; Litwiller et al., 1999; DasGupta and Fowler, 2002; Fernández, 2002). Urodynamic studies add mechanistic specificity: higher involuntary detrusor pressure during storage (PdetmaxIDC), reduced maximum cystometric capacity, and low compliance independently predict moderate–severe ED in MS cohorts with LUTD after accounting for age and disability, linking erectile impairment to quantifiable suprasacral neurogenic bladder physiology (Fragalà et al., 2014; Fragalà et al., 2015). Patient-reported and clinical measures move in parallel—worse IPSS and LUTS correlate with lower IIEF and sexual quality of life, and ED severity scales with EDSS—reinforcing a shared circuit substrate rather than independent sequelae (Balsamo et al., 2017; Nabavi et al., 2021; Bientinesi et al., 2022; Celik et al., 2013). Interventional observations are consistent with this coupling: neuromodulatory strategies targeting sacral circuitry, such as tibial nerve stimulation or related bioelectromagnetic approaches, produce concurrent improvements in bladder symptoms and erectile domains in small trials/series, supporting a modifiable sacral–suprasacral pathway in at least a subset of patients (Tomé et al., 2019; Giannopapas et al., 2024; Alzharani et al., 2024).

### 3.3 Autonomic–endothelial dysregulation as a downstream constraint

Erectile tumescence depends on endothelial nitric oxide availability, cGMP signaling, and dynamic cavernosal smooth muscle relaxation, which are sensitive to autonomic balance and systemic inflammatory tone (Ritchie and Sullivan, 2011). In MS, dysautonomia and immune activation plausibly reduce endothelial NO bioavailability, augment vasoconstrictor signaling through endothelin-1 and Rho-kinase, and blunt shear-mediated vasodilation, thereby increasing resting cavernosal tone and narrowing the hemodynamic response window (Ritchie and Sullivan, 2011; Zanin-Silva et al., 2021). Although direct vascular physiology specific to MS-ED is limited, therapeutic response patterns provide convergent evidence: randomized and observational studies show PDE5i improve erectile outcomes and related quality-of-life metrics in men with MS, implying that the endothelial–smooth muscle module remains at least partially druggable despite upstream neural injury (Fowler et al., 2005; Lombardi et al., 2010; Xiao et al., 2012). These findings do not negate central/spinal contributions but indicate that downstream endothelial function can constrain or facilitate expression of erectile capacity set by neural circuitry. Beyond MS-related neurogenic and autonomic injury, common systemic comorbidities such as diabetes mellitus, hypogonadism, hyperprolactinemia, and premature ejaculation may co-contribute to ED and should be considered in differential assessment (Rastrelli et al., 2025; Muneer et al., 2014).

### 3.4 Neuroinflammation, demyelination, and impaired plasticity

MS pathology—demyelination, axonal injury, and chronic inflammatory signaling—introduces conduction block and temporal dispersion along long tracts subserving genital afferents and autonomic efferents, lowering fidelity of psychogenic and reflexogenic erection pathways. Cytokine milieus can modulate neuronal excitability and nitric oxide synthase activity within arousal networks, amplifying deficits driven by structural lesions (Luo and Jiang, 2009). Conceptual and translational work on neurotrophin–Trk signaling suggests that compromised BDNF/NGF pathways may limit adaptive reorganization after central injury, potentially explaining lagging sexual function recovery relative to stabilization of gross disability and persistent abnormalities on genital SEPs despite partial preservation of nocturnal erections (Yang et al., 2001; Chaldakov et al., 2024). In a rat MS model, severe MS markedly reduced erectile function (lower ICPmax/MAP and nNOS expression) and produced ultrastructural cavernous nerve pathology—degeneration/demyelination of Schwann cells with preserved smooth muscle/endothelium—implicating neuropathic changes as a key mechanism of MS-related ED (Jiang et al., 2009). These mechanisms are supportive rather than definitive in MS-specific interventional terms but are compatible with observed electrophysiological and clinical patterns.

### 3.5 Psychosocial and relational modifiers within a constrained neurogenic framework

Depression, anxiety, fatigue, and relationship stress are prevalent in MS and independently associate with lower IIEF domains and diminished sexual quality of life, operating as modifiers of arousal appraisal and autonomic output rather than primary causes (Nabavi et al., 2021; Odabaş et al., 2018; Lew-Starowicz and Rola, 2014a; Bientinesi et al., 2022; Toljan and Briggs, 2024; Landtblom, 2006). Observational gradients—higher EDSS and longer disease duration associating with worse erectile function, with better outcomes when segmental reflexes and autonomic reserve are preserved—suggest that psychosocial factors act within ceilings imposed by lesion topology and tract integrity (Adamec et al., 2024; Hennessey et al., 1999). This layered model aligns with clinical practice were combining PDE5 inhibition or neuromodulation with mood and relational interventions tends to yield more consistent gains than single-modality strategies (Geng et al., 2023). Ejaculatory disturbances—including delayed, retrograde, or complete anejaculation—are frequently observed in men with MS, particularly in those with spinal cord involvement (Toljan and Briggs, 2024; Geng et al., 2023; Prévinaire et al., 2014; Trofimenko and Hotaling, 2016). Although spermatogenesis is usually preserved, impaired seminal emission or expulsion leads to functional infertility. These disturbances arise from neurogenic disruption of the sympathetic and parasympathetic pathways governing seminal emission and expulsion (Toljan and Briggs, 2024; Geng et al., 2023; Prévinaire et al., 2014; Trofimenko and Hotaling, 2016). Beyond its biological consequences, infertility imposes substantial psychosocial burdens, amplifying sexual distress and relationship strain. According to the 2024 International Consultation on Sexual Medicine (ICSM) consensus, infertility in men with MS most often results from ejaculatory dysfunction—particularly anejaculation or retrograde ejaculation. Early fertility assessment and, when appropriate, penile vibratory stimulation or electroejaculation are recommended interventions (Elliott et al., 2025).

Another underappreciated dimension concerns the partner's evolving role in advanced or long-standing MS. As disability progresses, partners may increasingly assume caregiving responsibilities, blurring the boundaries between caregiver and intimate partner. This shift frequently diminishes mutual desire, alters intimacy, and reshapes sexual behavior. The ICSM 2024 review further emphasizes that neurological disability disrupts intimacy dynamics and underscores the need for partner involvement in psychosexual counseling and rehabilitation (Elliott et al., 2025).

### 3.6 Integrative synthesis

Collectively, available evidence supports a convergent neurogenic–vasculogenic framework. Insular–limbic and brainstem network injury reduces arousal-related autonomic drive and the integration of genital afferent input; suprasacral demyelination degrades descending control of sacral autonomic/somatic nuclei, manifesting as coupled neurogenic bladder and ED; autonomic–endothelial dysregulation narrows cavernosal vasodilatory capacity; and inflammatory–trophic disturbances likely limit compensatory plasticity. The coherence across lesion mapping, electrophysiology, urodynamics, disability gradients, and pharmacologic responsiveness supports biological plausibility without overstating causality. Where evidence is indirect (e.g., endothelial pathways), inferences are anchored by treatment responsiveness and established ED pathophysiology, while MS-specific mechanistic gaps are noted.

## 4 Potential MS-targeted strategies for managing ED

### 4.1 Diagnostic stratification: make the cause explicit before treating

**FIGURE 2:**
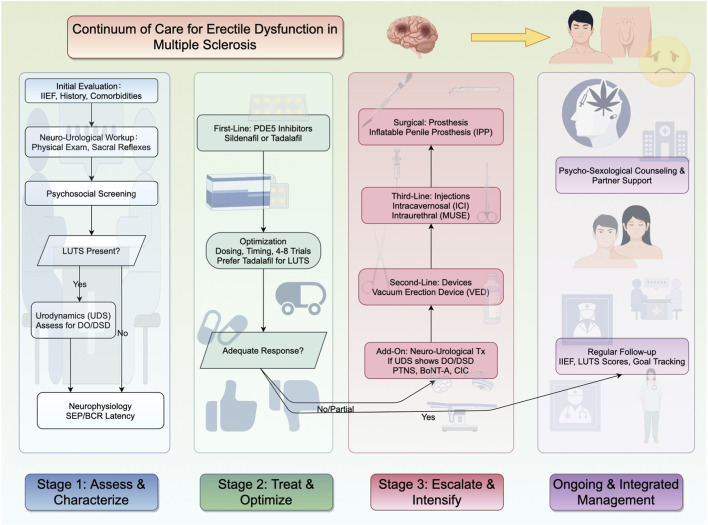
Continuum of care for ED in MS: A stage-based, multidisciplinary algorithm. Figure outlines a stepwise, multidisciplinary pathway for evaluating and managing ED in MS. Stage 1 emphasizes assessment and characterization using IIEF, medical history, comorbidities, neuro-urological examination (physical exam, sacral reflexes), and psychosocial screening; if LUTS are present, UDS is recommended to assess DO/DSD, with optional neurophysiology testing (SEP and BCR latency). Stage 2 involves a trial of first-line PDE5 inhibitors (sildenafil or tadalafil), with careful adjustment of dose and timing over at least 4–8 attempts, as recommended by ED guidelines. Tadalafil may be considered when LUTS are also present. If response is adequate, therapy continues with monitoring; otherwise, proceed to Stage 3. Stage 3 escalates care with add-on neuro-urological treatments guided by UDS findings (e.g., PTNS, BoNT-A, CIC), followed by second-line devices such as VED, third-line options including ICI or MUSE, and, when appropriate, surgical implantation of an IPP. Across all stages, integrated management includes psycho-sexological counseling, partner support, and regular follow-up with IIEF and LUTS scores to track goals. Abbreviations: ED, erectile dysfunction; MS, multiple sclerosis; IIEF, International Index of Erectile Function; LUTS, lower urinary tract symptoms; UDS, urodynamics; DO, detrusor overactivity; DSD, detrusor sphincter dyssynergia; SEP, somatosensory evoked potentials; BCR, bulbocavernosus reflex; PDE5i, phosphodiesterase type 5 inhibitor(s); PTNS, percutaneous tibial nerve stimulation; BoNT-A, onabotulinumtoxinA; CIC, clean intermittent catheterization; VED, vacuum erection device; ICI, intracavernosal injection; MUSE, medicated urethral system for erection; IPP, inflatable penile prosthesis.

Management should begin by aligning therapy to demonstrable dysfunction rather than symptoms alone. Observational gradients show that greater disability correlates with worse erectile function independently of age and disease duration, defining a neurological “ceiling” that frames expectations and sequencing (Adamec et al., 2024), while early case–control work confirms an overall higher risk of sexual dysfunction in MS versus controls (Zorzon et al., 1999; Denys et al., 2013; Donzé and Hautecoeur, 2009). Bedside examination anchors localization: clustering of brainstem and pyramidal signs supports suprasegmental and long-tract involvement in arousal/autonomic drive, whereas preserved cremasteric and bulbocavernosus reflexes indicate residual sacral circuitry and therapeutic headroom (Betts et al., 1994; Hennessey et al., 1999; Litwiller et al., 1999; DasGupta and Fowler, 2002). In patients with discordant symptoms—such as preserved nocturnal penile tumescence but intercourse failure—neurophysiology helps resolve whether recruitment failure is central or peripheral; abnormal genital somatosensory evoked potentials and prolonged bulbocavernosus reflex latency identify impaired afferent conduction and central integration despite intact end-organ generators, and this pattern justifies a focused attempt to pharmacologically recruit cavernosal smooth muscle while setting realistic expectations (Valleroy and Kraft, 1984; Yang et al., 2001; Kirkeby et al., 1988a; Kirkeby et al., 1988b). Where lower urinary tract symptoms coexist, urodynamics quantify a suprasacral pattern—elevated storage detrusor pressures during involuntary contraction, reduced maximum cystometric capacity, and low compliance—that independently predict moderate–severe ED after adjusting for age and disability; these measurements both explain limited hemodynamic recruitment and nominate candidates for sacral-targeted neuromodulation (Fragalà et al., 2014; Fragalà et al., 2015; Litwiller et al., 1999; DasGupta and Fowler, 2002; Fernández, 2002). In parallel, brief screening for depression, anxiety, fatigue, and relationship stress is essential because these factors independently depress IIEF domains and sexual quality of life and can blunt responses to biomedical therapy if unaddressed (Nabavi et al., 2021; Odabaş et al., 2018; Lew-Starowicz and Rola, 2014a; Lew-Starowicz and Rola, 2014b; Bientinesi et al., 2022; Celik et al., 2013). Endocrine and metabolic screening (including serum testosterone, glycemia, and prolactin) is advisable to rule out non-neurological contributors (Rastrelli et al., 2025). This layered appraisal provides a concrete basis to select, combine, and pace interventions in a mechanism-concordant way (Figure 2). Recent ICSM reports have emphasized the multidimensional evaluation of male sexual dysfunction, integrating neurogenic, endocrine, and psychosocial perspectives, which align with our framework (Rastrelli et al., 2025; Elliott et al., 2025).

### 4.2 PDE5-anchored therapy: start with oral agents and test the end-organ window

MS-related ED is multifactorial. PDE5 inhibitors are commonly recommended as a first-line therapy for MS-associated ED, but current evidence suggests only modest efficacy, and recommendations should be tailored to individual patient characteristics and response (Prévinaire et al., 2014; Moussa et al., 2021; Francomano et al., 2017; de Sèze and Gamé, 2014). First-line therapy for MS-associated ED may benefit from targeting the endothelial–smooth muscle module, as residual end-organ pathways can sometimes be recruited despite upstream neurogenic injury. Randomized controlled and observational studies in MS populations have shown modest average improvements in erectile function and patient satisfaction with agents such as sildenafil and tadalafil, suggesting that cGMP-mediated vasodilation remains a therapeutic option in appropriately selected patients. However, overall effect sizes are moderate, and individual responses vary substantially (Fowler et al., 2005; Lombardi et al., 2010; Xiao et al., 2012; Lombardi et al., 2012; Zhang et al., 2011). Practical delivery generally requires adequate dosing and sufficient trial exposure—such as titrating sildenafil from 50 to 100 mg or tadalafil from 10 to 20 mg, with at least four to eight well-timed attempts, as recommended in general ED management guidelines—before designating treatment as non-responsive. In men with concurrent storage-phase LUTS, tadalafil may provide incremental symptom relief that could facilitate better sexual outcomes, although direct head-to-head comparisons in MS populations remain limited (Balsamo et al., 2017; Bientinesi et al., 2022). Nonresponse is generally determined in clinical practice only after at least 4–8 properly timed, on-label attempts at adequate doses (e.g., sildenafil 50–100 mg; tadalafil 10–20 mg), with appropriate attention to meal timing, alcohol intake, and sufficient sexual stimulation. When response is suboptimal, considerations should include verifying timing relative to meals, reviewing for concomitant alpha-blockers or antihypertensives that may contribute to autonomic hypotension, and assessing for urodynamic factors such as high-pressure storage or poor bladder compliance, which may reduce cavernosal inflow. The pharmacologic rationale aligns with general erectile pathophysiology: reduced nitric oxide bioavailability and elevated endothelin-1/Rho-kinase tone can narrow the hemodynamic response window. This underpins the clinical practice of optimizing vascular risk and sleep quality in MS-ED management, while acknowledging that causality in the MS setting is not absolute. (Ritchie and Sullivan, 2011; Zanin-Silva et al., 2021; Vodušek, 2009).

### 4.3 Sacral-targeted neuromodulation and LUTD optimization: use urodynamics to guide when to add

When diagnostics show the canonical suprasacral pattern—detrusor overactivity, detrusor–sphincter dyssynergia, and reduced compliance—modulating sacral networks can extend the recruitment window for erections and simultaneously improve bladder control. Posterior tibial nerve stimulation and related bioelectromagnetic approaches, commonly delivered as weekly induction courses with maintenance, produce parallel gains in LUTS and erectile indices in MS cohorts, consistent with a shared sacral circuitry that remains biasable despite demyelination (Tomé et al., 2019; Giannopapas et al., 2024; Alzharani et al., 2024). Patients with preserved segmental reflexes and measurable storage-phase activity tend to respond better, reflecting residual network integrity and providing a rational selection criterion (Betts et al., 1994; Fragalà et al., 2015; Litwiller et al., 1999). In tandem, targeted management of neurogenic LUTD—antimuscarinics or beta-3 agonists for detrusor overactivity, intradetrusor botulinum toxin for refractory high-pressure storage, and clean intermittent catheterization for incomplete emptying—reduces sympathetic overdrive, paradoxical pelvic floor co-contraction, and sleep fragmentation, all of which destabilize erectile performance if untreated (Balsamo et al., 2017; Fragalà et al., 2015; Bientinesi et al., 2022; DasGupta and Fowler, 2002; Fernández, 2002). In practice, PDE5 inhibitors remain the anchor, neuromodulation is added for partial responders with urodynamic constraints, and LUTD therapy is maintained to stabilize the physiologic milieu required for consistent erections.

### 4.4 Escalation and integrated psychosexual care: achieve function within a lesion-defined ceiling

When oral therapy and neuromodulation are insufficient, escalation should follow established ED pathways while accounting for MS-specific constraints such as dexterity, spasticity, and bladder routines. Vacuum erection devices offer a neuro-independent solution whose effectiveness hinges on training and partner engagement; intracavernosal alprostadil with or without papaverine/phentolamine, or intraurethral alprostadil, directly relaxes cavernosal smooth muscle and can provide predictable rigidity when autonomic drive is limited, though clinicians should anticipate and plan for injection technique and spasticity management; for refractory cases, penile prosthesis offers durable restoration of penetrative function provided perioperative planning addresses neurogenic bladder care and infection risk (Fowler et al., 2005; Lombardi et al., 2010; Xiao et al., 2012; Li et al., 2020). In men with neurological disease including MS who failed first-line ED treatments, penile prosthesis implantation yielded high, durable satisfaction (patient ∼75/100; partner ∼67/100) and preserved device handling over ∼6 years, despite a higher infection rate than in the general ED population (Xardel et al., 2021). These findings support considering penile prosthesis as a viable option for MS-related ED after medical/device therapy failure. Throughout escalation, integrated psychosexual care—routine brief screening, timely cognitive-behavioral or sex therapy, and couple-focused interventions—targets performance anxiety, attentional bias, and dyadic stress that independently depress IIEF domains and can attenuate biomedical gains; framing goals within a neurologically constrained ceiling supports adherence and satisfaction without inflating expectations (Liu et al., 2018; Nabavi et al., 2021; Odabaş et al., 2018; Lew-Starowicz and Rola, 2014a; Bientinesi et al., 2022; Fode et al., 2012). Longitudinal follow-up should reassess erectile function, LUTS, and quality of life on a fixed cadence, adjust PDE5 dosing, repeat urodynamics when symptoms evolve, and revisit neuromodulation candidacy as reflexes and storage pressures change. Lesion mapping that implicates insular hubs and urodynamic signatures of suprasacral dysfunction predict more severe ED and reduced pharmacologic headroom, yet multimodal care achieves meaningful gains across strata; disability level remains a strong correlate of erectile outcomes, but improvements are common with the combined pathway described here (Winder et al., 2018; Adamec et al., 2024; Fragalà et al., 2014; Fragalà et al., 2015; Tomé et al., 2019; Giannopapas et al., 2024; Alzharani et al., 2024). Finally, while disease-modifying therapies stabilize relapse risk and disability trajectories, their effects on sexual function are heterogeneous and incompletely characterized; ED changes should not be attributed to DMTs without corroborating neurophysiology or urodynamics, though inflammatory and neurotrophic milieus likely influence recovery timelines and the persistence of SEP abnormalities (Yang et al., 2001; Luo and Jiang, 2009; Chaldakov et al., 2024).

## 5 Conclusion and future outlooks

MS-related ED is best understood as the convergence of neurogenic injury, lower urinary tract dysfunction, endothelial–smooth muscle limits, and psychosocial load (Bientinesi et al., 2022; DasGupta and Fowler, 2002; Fernández, 2002). The most consistent epidemiologic signal is a disability–erectile function gradient independent of age and disease duration, which sets a lesion-defined ceiling for prognosis and sequencing (Zorzon et al., 1999; Adamec et al., 2024). Mechanism-aware diagnostics—bedside localization (brainstem/pyramidal signs, sacral reflex integrity), targeted neurophysiology (genital SEP, BCR latency), and urodynamics (storage pressure, capacity, compliance, dyssynergia)—move care beyond symptom labels to actionable physiology (Betts et al., 1994; Valleroy and Kraft, 1984; Yang et al., 2001; Kirkeby et al., 1988a; Kirkeby et al., 1988b; Fragalà et al., 2014; Fragalà et al., 2015; Litwiller et al., 1999). This enables a rational pathway: anchor with PDE5 inhibitors to recruit end-organ reserve; add sacral-focused neuromodulation and structured LUTD therapy when urodynamics show suprasacral loading; and escalate to devices, injections, or prosthesis when neurogenic constraints limit pharmacologic headroom (Tomé et al., 2019; Giannopapas et al., 2024; Alzharani et al., 2024; Fowler et al., 2005; Lombardi et al., 2010). Psychosexual care should be integrated throughout, given the independent impact of mood, anxiety, fatigue, and dyadic stress on IIEF domains and adherence (Nabavi et al., 2021; Odabaş et al., 2018; Lew-Starowicz and Rola, 2014a; Lew-Starowicz and Rola, 2014b; Celik et al., 2013). Vascular/endothelial contributors that narrow the hemodynamic response window—reduced nitric oxide bioavailability and heightened endothelin-1/Rho-kinase tone—support risk-factor optimization in parallel with neurogenic care without overstating causality in MS (Ritchie and Sullivan, 2011; Zanin-Silva et al., 2021).

Current evidence is directionally robust but methodologically uneven: many studies are small, single-center, and heterogeneous in outcome measures; MS-specific head-to-head trials of PDE5 strategies are scarce; neuromodulation protocols vary and are inconsistently sham-controlled; neurophysiology and urodynamic criteria are underused in enrollment and response definitions; DMT–sexual function interactions remain insufficiently resolved; and psychosexual arms are frequently underpowered (Tomé et al., 2019; Giannopapas et al., 2024; Alzharani et al., 2024; Fowler et al., 2005; Lombardi et al., 2010; Chaldakov et al., 2024). Next steps should prioritize standardized phenotyping (EDSS strata, lesion topology including brainstem/insular hubs, reflex status, genital SEP/BCR, and urodynamic thresholds), mechanism-stratified randomized trials that test additive sequencing (e.g., PDE5 ± tibial neuromodulation in high-pressure storage phenotypes; early intracavernosal therapy when reflexes are absent), protocol optimization for neuromodulation with objective physiologic endpoints, and incorporation of wearable/autonomic and home NPT monitoring to link day-to-day physiology with response (Winder et al., 2018; Fragalà et al., 2014; Fragalà et al., 2015). Embedding sexual function endpoints into prospective DMT cohorts and co-primary designs that pair biomedical and psychosexual interventions will strengthen causal inference and implementation (Nabavi et al., 2021; Lew-Starowicz and Rola, 2014a; Luo and Jiang, 2009; Bresch et al., 2025). With these advances, care can shift from broadly effective to precisely targeted, delivering durable improvements in sexual function and quality of life for people living with MS.

Although current evidence delineates several plausible neurogenic–vascular pathways, most data remain cross-sectional and lack causal validation. The field still relies heavily on extrapolation from general ED research. Integrating mechanistic biomarkers, longitudinal imaging, and partner-centered outcomes could provide transformative insight. Future work should challenge current assumptions that MS-ED is purely neurogenic and test whether metabolic or inflammatory modulation could restore autonomic–endothelial balance.
